# Therapeutic Applications of Functional Nanomaterials for Prostatitis

**DOI:** 10.3389/fphar.2021.685465

**Published:** 2021-05-28

**Authors:** Chun-Ping Liu, Zi-De Chen, Zi-Yan Ye, Dong-Yue He, Yue Dang, Zhe-Wei Li, Lei Wang, Miao Ren, Zhi-Jin Fan, Hong-Xing Liu

**Affiliations:** ^1^ Department of Urology, Guangzhou Institute of Urology, Guangdong Key Laboratory of Urology, The First Affiliated Hospital of Guangzhou Medical University, Guangzhou Medical University, Guangzhou, China; ^2^ The Second Affiliated Hospital of Guangzhou University of Chinese Medicine, Guangzhou, China; ^3^ Department of Interventional Radiology, Cancer Center, Guangdong Provincial People’s Hospital, Guangdong Academy of Medical Sciences, South China University of Technology, Guangzhou, China; ^4^ State Key Laboratory of Quality Research in Chinese Medicine, Institute of Chinese Medical Sciences, University of Macau, Macau, China; ^5^ Guangdong Provincial People’s Hospital, School of Medicine, South China University of Technology, Guangzhou, China

**Keywords:** prostatitis, functional nanoparticle, inflammatory microenvironment, engineering strategy, reactive oxygen species

## Abstract

Prostatitis is a common disease in adult males, with characteristics of a poor treatment response and easy recurrence, which seriously affects the patient’s quality of life. The prostate is located deep in the pelvic cavity, and thus a traditional infusion or other treatment methods are unable to easily act directly on the prostate, leading to poor therapeutic effects. Therefore, the development of new diagnostic and treatment strategies has become a research hotspot in the field of prostatitis treatment. In recent years, nanomaterials have been widely used in the diagnosis and treatment of various infectious diseases. Nanotechnology is a promising tool for 1) the accurate diagnosis of diseases; 2) improving the targeting of drug delivery systems; 3) intelligent, controlled drug release; and 4) multimode collaborative treatment, which is expected to be applied in the diagnosis and treatment of prostatitis. Nanotechnology is attracting attention in the diagnosis, prevention and treatment of prostatitis. However, as a new research area, systematic reviews on the application of nanomaterials in the diagnosis and treatment of prostatitis are still lacking. In this mini-review, we will highlight the treatment approaches for and challenges associated with prostatitis and describe the advantages of functional nanoparticles in improving treatment effectiveness and overcoming side effects.

## Background

Prostatitis is one of the most common urogenital diseases and mainly manifests as hypogastrium, perineum, scrotum, urethra and penis pain, and even bladder irritation, seriously affecting the patient’s quality of life (Krieger et al., 2008; Brede and Shoskes, 2011; Kogan et al., 2018). According to statistics, approximately half of males have ever suffered from prostatitis, and prostatitis outpatient services account for approximately 25% of services provided by urology clinics. Prostate cancer and benign prostatic hyperplasia mainly occur in older males, while prostatitis occurs in males of all ages, especially in young and middle-aged males (Drake et al., 2021). It is the third most common urinary disease in males (Khan et al., 2017).

Prostatitis is mainly divided into 2 class I acute bacterial prostatitis, class II chronic bacterial prostatitis, class III chronic prostatitis/chronic pelvic pain syndrome, and class IV asymptomatic inflammatory prostatitis (Krieger et al., 1999). In addition, chronic prostatitis/chronic pelvic pain syndrome accounts for 90-95% of prostatitis cases (Sharma and Kumar, 2021). In 2008, the National Institutes of Health (NIH)-affiliated National Institute of Diabetes, Digestive and Kidney Disease established the Map Research Network to guide researchers in more disciplines to participate in collaborative research on chronic pelvic pain and to update and improve its definition and treatment standards. Currently, the pathogenic factors causing chronic prostatitis in the clinic are controversial. Traditional treatments for prostatitis include antibiotics, antioxidants, and surgery (Vahlensieck et al., 2013; Ihsan et al., 2018). Many patients have turned to alternative therapies because of the limited effectiveness of traditional therapies and recurrence. In recent years, physical therapies for prostatitis have included biofeedback, hyperthermia, and magnetic therapy, but the efficacy and side effects are controversial (Hu et al., 2019; Birowo et al., 2020). Therefore, studies exploring the etiology and pathogenesis of prostatitis and identifying new strategies to improve its therapeutic effectiveness are needed. This review highlights the treatment approaches for and challenges associated prostatitis and describes the advantages of functional nanoparticles in improving treatment effectiveness and overcoming side effects.

## Main Factors Causing Prostatitis

**FIGURE 1:**
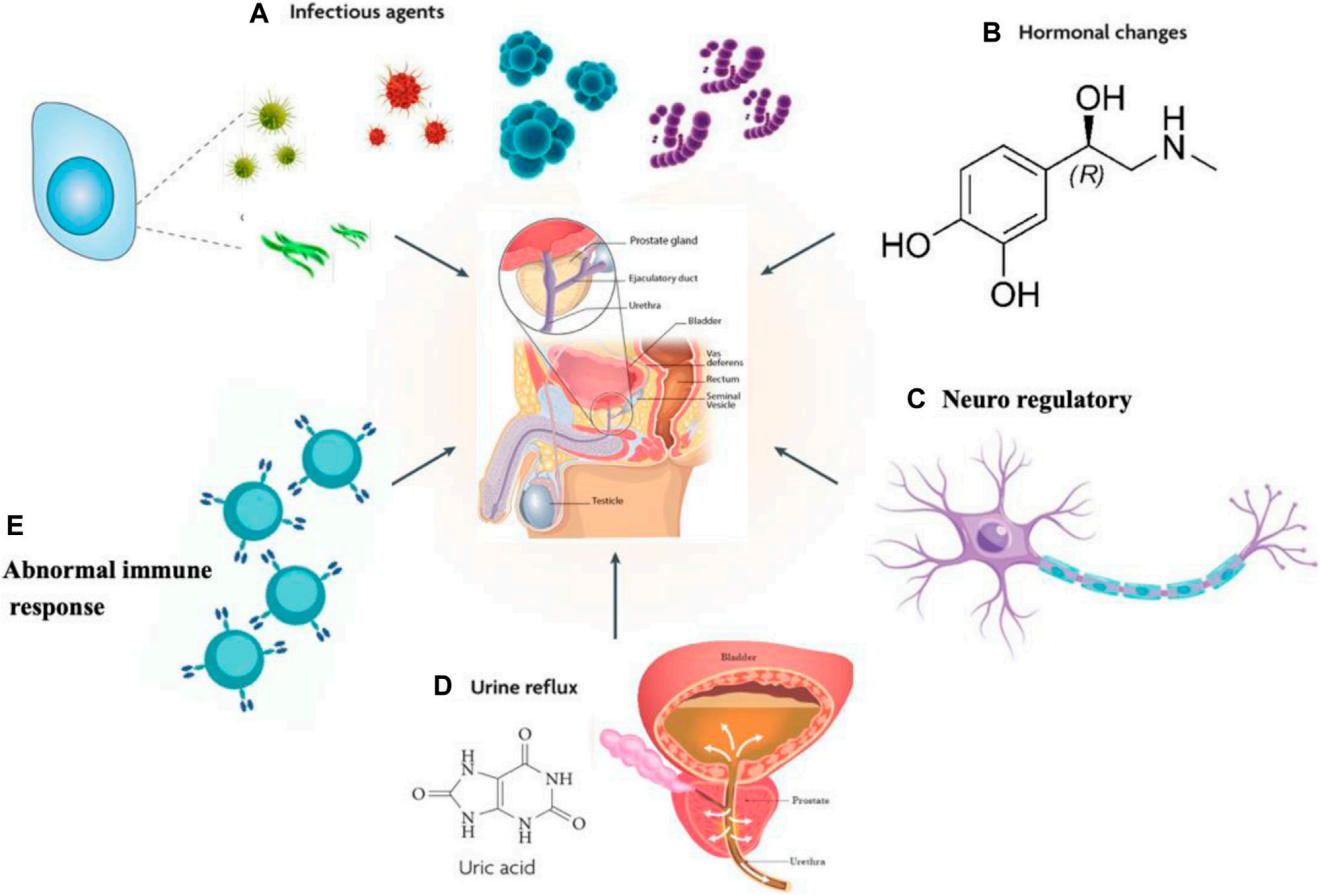
Possible causes of prostate inflammation. (**A**) Pathogen infection. (**B**) Sex hormone imbalance. (**C**) Urination dysfunction. (**D**) Neuroregulatory mechanisms. (**E**) Abnormal immune response.

The pathogenesis of prostate disease is complex with numerous influencing psychological factors, including pathogen infection, sex hormone imbalance, urination dysfunction, inflammation, and abnormal immune response (Sharp et al., 2010), among which the inflammatory response is the key pathological mechanism of prostatitis, and the inflammatory microenvironment determines the process of prostatitis, (Crocetto et al., 2020; Huang et al., 2020) as schematically depicted in Figure 1.

### Pathogen Infection

Viruses, fungi, bacteria and other pathogenic microorganisms can cause prostatitis, and bacterial infection is an important pathogenic factor causing prostatitis (Delcaru et al., 2016; Khan et al., 2017). Most of the pathogens detected in patients with prostatitis are gram-negative bacteria, and 60% of the bacteria are *Escherichia coli* (Barzelai and Whittem, 2017; Zhao et al., 2019; Su et al., 2020). In anti-infection treatment, because the pathogenic bacteria increase or exert an inhibitory effect on the defense function of the patient, pathogenic bacteria exist for a long time and cannot be eradicated (Benway and Moon, 2008). Bacterial infection may be the trigger rather than the cause of the clinical syndrome.

### Sex Hormone Imbalance

The prostate is a sex accessory organ, and pathological changes in the prostate and the progression of prostatitis are closely related to sex hormones and their receptors (Letkiewicz et al., 2020). In addition, prostate gland lesions and the occurrence and development of prostatitis are closely related to sex hormones and their receptors, and a sex hormone imbalance is the main reason for class IIIB prostatitis (Lan et al., 2017).

### Urination Dysfunction

Uric acid is filtered through the glomerulus and is the product of nucleic acid decomposition, cell metabolism and purine metabolism in the body. Most urate is reabsorbed through the proximal convolution tubule, but an accumulation of urate crystals in the tissue leads to an inflammatory reaction that produces high-frequency contraction and spasm of the urethral sphincter. These changes cause an imbalance in bladder detrusor and sphincter synergism or bladder outlet obstruction and urine reflux.

### Neuroregulatory Mechanisms

Neuroregulatory mechanisms are closely related to prostatitis. In patients with prostatitis (Park et al., 2015; Shulyak et al., 2019), inflammation is stimulated and may cause long-term nervous system damage that result in, clinical symptoms with a spinal nerve segmental dominance. Prostate pain may be the cause of spinal nerve segmental secondary lesions. Some prostatitis pain may be caused by constant pain in spinal nerve segmental nerves, but scholars have also indicated that prostatitis pain may be due to the abnormal state of the chronic neuroregulatory mechanism caused by multiple factors or a single cause, which may be related to spinal cord glial cells or spinal cord nerve cells (Shih et al., 2020).

### Abnormal Immune Response

Relevant studies have suggested that prostatitis is likely an autoimmune disease (Motrich et al., 2007). People with normal immune function generally do not experience inflammation after an infection, while those with low immune function are prone to inflammation. Some scholars also proposed that the prostate is an immune organ with more than 90% T lymphocytes, which exist in the epithelial stromal area of the gland, along with a small number of other inflammatory cells (Motrich et al., 2020). T lymphocytes produce IFN-γ and stimulate the production of IL-15 in the prostate, and this paracrine signaling is the cause of chronic inflammation (Handisurya et al., 2001). Both prostatic epithelial cells and stromal cells express cytokine receptors, participate in local immune regulation as anti-inflammatory presenting cells (Carlo et al., 2007; Penna et al., 2009; Fibbi et al., 2010; De Nunzio et al., 2011), and secrete pro-inflammatory cytokines such as IL-1α, IL-1β and IL-6 (Kramer et al., 2003; Beadling and Slifka, 2006; Magri et al., 2019). Prostatitis is considered an autoimmune disease (Li et al., 2019).

## Challenges in the Clinical Treatment and Diagnosis of Prostatitis

Currently, ideal treatment and diagnostic methods for prostatitis are still lacking, and thus new drug delivery systems and diagnostic strategies for prostatitis are urgently needed.

### Challenges in the Treatment of Prostatitis

The etiology of prostatitis is unclear due to the numerous symptoms with no specificity (Verze et al., 2016). In recent years, some experts have proposed the concept of prostatitis syndrome, a clinical syndrome with different etiologies, clinical manifestations, disease processes and responses to treatment (Ramakrishnan and Salinas, 2010). Antibiotics, nonsteroidal anti-inflammatory analgesics and alpha-blockers are used in the traditional clinical treatment of prostatitis (Xiong et al., 2021). In addition, pharmacological treatments remain largely ineffective due to the difficulty in penetrating the prostatitis microenvironment. Prostatitis is characterized by inflammatory hyperplasia, a high pH, bacterial accumulation and a disruption of the blood-prostate barrier (El Meliegy and Torky, 2015). These four characteristics and properties are analyzed in the remainder of the article.

#### Inflammatory Hyperplasia

Prostatitis is accompanied by inflammatory hyperplasia, leading to prostatic hyperplasia and edema, prostate duct stenosis or obstruction caused by pressure in the gland, and the blood circulation barrier obstructs the entry of drugs (Ravindran et al., 2020). At the same time, inflammatory exudates extravasate around the prostate due to high pressure, causing or exacerbating symptoms of pelvic and urinary tract irritation.

#### High pH

In addition, prostatitis increases the pH of the prostate and decreases drug dispersion, and the concentration of drug that penetrates the prostatic canal, acini and prostatic fluid is insufficient. Recurring episodes of prostatitis lead to the formation of calcified plaques in the prostate.

#### Bacterial Accumulation

Bacteria accumulate inside or on the surface of calcified plaques (Dibb et al., 2001), exist and multiply sustainably under protective biofilms. Calcified spots can develop into stones, which may block the prostate gland duct and induce infection. Therefore, calcified spots and stones are important factors affecting the effectiveness of prostatitis treatment, leading to repeated attacks.

#### Blood-Prostate Barrier

Rectal administration is one of the most common methods used in prostate treatment, but some drugs are unable to pass through the blood-prostate barrier and do not reach effective therapeutic concentrations in the prostate tissue and acinus. (El Meliegy and Torky, 2015) Direct injection into the prostate solves the problem of the prostate anatomical barrier, but invasive treatment easily causes damage to the nerve and vascular tissues of the perineum and aggravates local inflammation.

### Challenges in the Diagnosis of Prostatitis

The NIH classifies prostatitis into four subtypes (Sharma and Kumar, 2021), and the main cause of type I and II prostatitis is pathogen infection. According to the type of pathogen, the choice of appropriate antibiotics results in a better treatment effect. Type IV prostatitis is difficult to detect due to a lack of clinical symptoms, relevant pathogenesis and treatment studies. Among the CP/CPPS, is the most common, accounting for more than 90% of chronic prostatitis cases. (Holt et al., 2016) The diagnostic criteria are that the patient has persistent or recurrent pain in the pelvic area for at least 3 of the past 6°months. However, the definition of prostatitis is still relatively vague, the classification is complex, the diagnostic method is also quite controversial, and reliable physical and chemical indicators are lacking. No unified standard for the clinical diagnosis and evaluations of the curative effect are available, and the curative is generally difficult to evaluate and analyze (Coker and Dierfeldt, 2016).

## Application of Functionalized Nanomaterials in Prostatitis

Many pathogenic factors contribute to prostatitis (Delcaru et al., 2016), and lesions induced by different factors require different detection and treatment methods, which undoubtedly increases the difficulty of diagnosing and treating prostatitis. At the same time, urethral inflammation has a long disease course, and traditional diagnostic and treatment methods are invasive, which will exert a certain effect on the patient’s body and mind (Mangir and Chapple, 2020). For example, the most commonly used mode in clinical practice, rectal administration, may damage the intestinal mucosa due to unstable drug absorption. Therefore, the treatment of prostatitis requires good imaging performance, strong compatibility and high universality of imaging technology, and a high bioutilization of pharmaceutical preparations.

The inflammatory response is the core pathological mechanism of prostatitis and the key link affecting the disease process (Motrich et al., 2018). Methods to effectively alleviate the inflammatory microenvironment are the key to improving the clinical efficacy of prostatitis treatments. In addition, prostatitis is often accompanied by a microbial infection. For prostatitis caused by a microbial infection (Kogan et al., 2018), treatment with anti-infectious agents is the most direct and effective method. Inflammation and infection are also major diagnostic indicators of urethral inflammation, including prostatitis.

**FIGURE 2:**
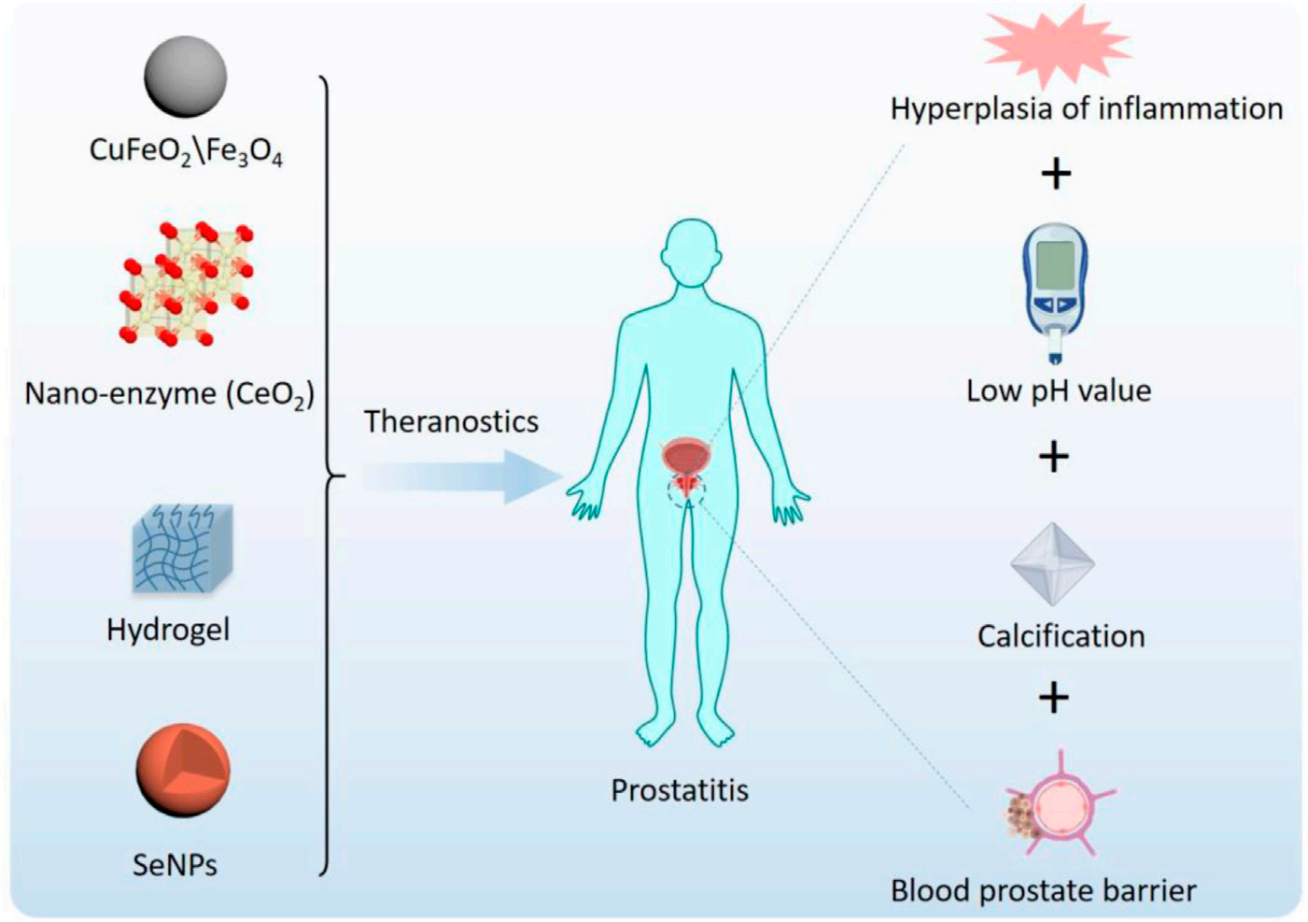
Smart nanoparticles for prostatitis.

Nanotechnology refers to the study and application of materials at the nanoscale, and its application in the medical field is called nanomedicine (Richardson and Caruso, 2020). Advances in nanotechnology have facilitated the development of delivery systems to treat prostate-related disorders. Advantages of nanocarrier preparations include the combination of a variety of drugs, including biomacromolecule drugs; reduced degradation of unstable drugs for slow and controlled release; and increased residence time of relevant drugs to avoid frequent injections and meet the needs of prostatitis treatment (Thakur and Agrawal, 2015; Liu et al., 2020; Liu et al., 2020; Wang et al., 2020; Lin et al., 2021; Liu et al., 2021). More importantly, the modular design and preparation characteristics of nanotechnology endow nanomaterials with intelligent characteristics (van der Meel et al., 2019). Smart NPs are designed to respond to environmental or external stimuli that trigger drug release after passive or active accumulation, as schematically depicted in Figure 2.

Nanotechnology is a powerful tool for developing new treatments and diagnoses for prostatitis and is expected to continue to grow in the future. In recent years, a number of nanomaterials with anti-inflammatory and antimicrobial properties have emerged,including CuFeO_2_ and Fe_3_O_4_ NPs, nanohydrogels, photosensitive H_2_-generated nanosystems, and polydopamine nanoparticles (Salari et al., 2018; Yu et al., 2018; Zhao et al., 2018; Antonoglou et al., 2019; Zhang et al., 2019). Nanomaterials with anti-inflammatory and anti-infective properties show good application prospects in the treatment of prostatitis. We will summarize the applications of functionalized nanomaterials in prostatitis and evaluate the advantages and disadvantages (Table 1).

**TABLE 1:** The application of functionalized nanomaterials in prostatitis.

Nanocarrier	Therapeutic strategy	Model	Effective constituent	Advantages	Disadvantages	Refs.
silver NPs	Antimicrobial for multidrug-resistant bacteria	Urinary tract infections	Silver	Inhibits biofilm formation, inhibits the growth of UTI-causing pathogens. Inhibits multidrug-resistant bacteria	Metabolic toxicity	(Divya et al., 2019 ^*)*^ (Lopez-Carrizales et al., 2018) (Maharubin et al., 2019 ^),^ (Al-Ansari et al., 2020 ^)^(El-Batal et al., 2019 ^)^
sulfur NPs	Antimicrobial	Urinary tract infections	Sulfur	Use as an antibacterial agent alone or in combination with antibiotics to exert synergistic effects	Metabolic toxicity	(Paralikar et al., 2019 ^**)**^
Zinc oxide NPs	antioxidant activity and antibacterial activity	Urinary tract infections	Zinc oxide	ZnO NPs displayed antibacterial activities and moderate antioxidant potential.	none	(Santhoshkumar et al., 2017; Chandra et al., 2019; Hosseini et al., 2019; Abd Elkodous et al., 2020)
Extracellular vesicles	Anti-inflammatory	chronic prostatitis	Extracellular vesicles	Ameliorates chronic pelvic pain, improves voiding dysfunction, suppresses inflammatory reactions, and facilitates prostatic tissue repair.	preparation is relatively complicated and the active ingredients are complex	(Peng et al., 2021 ^**)**^
Nanoparticle-conjugated Autoantigen Peptide T2	Anti-inflammatory	Autoimmune prostatitis	Autoantigen Peptide T2	Ameliorates the manifestations of CP/CPPS that will improve the effectiveness of therapeutic approaches.	autoimmune risk	(Cheng et al., 2019 ^**)**^
Selenium NPs	Antimicrobial	Urinary tract infection	Selenium	Increased percentage of biofilm. Efficient inhibition of *S. aureus, P. aeruginosa,* and *E. coli*.	none	(El-Sayyad et al., 2020 ^**)**^
copper NPs	Antioxidant and antibacterial	Urinary tract infection-causing pathogens	Copper	Proved to effectively kill or significantly inhibit the activity of urinary tract infection-causing pathogens and exhibits excellent antioxidant activity.	Metabolic toxicity	(Malarkodi and Rajeshkumar, 2017; Al-Enizi et al., 2018)
PLGA nanoparticles	Antimicrobial	Urinary tract infections	Trimethoprim	No effects on metabolism and good histocompatibility	One function of the carrier	(Brauner et al., 2020 ^**)**^

### The Application of Functionalized Nanomaterials in Prostatitis

#### Inorganic Nanomaterials

Inorganic nanomaterials have been widely used in biomedical fields because of their easy availability and stable properties. Inorganic nanomaterials generally refer to the incorporation of metal and nonmetal elements, metal oxides, salts and other components into nanoparticles alone or in combination (Rao et al., 2007). These nanomaterials have different physical and chemical properties due to their different compositions and structures. Thus, different inorganic nanomaterials have different applications (Liang et al., 2014). In UTI (including prostatitis), inorganic nanomaterials are mainly used in the scenarios described below.

Inflammation is associated with oxidative stress and can be alleviated by antioxidants (Czarny et al., 2018). A variety of inorganic nanomaterials have been found to possess antioxidant activity. Iron nanoparticles, such as Fe_3_O_4_ nanoparticles, reduce oxidative pressure by catalyzing the degradation of H_2_O_2_ (Alavi and Karimi, 2019). Fe_3_O_4_ is also considered a magnetic nanoparticle with good biocompatibility and anti-inflammatory activity (Xie et al., 2019). Fe_3_O_4_ nanoparticles have been combined with various anti-inflammatory drugs as a new strategy for the treatment of prostatitis (Kojima et al., 2018). Nanoparticles composed of another metal oxide, zinc oxide, are also widely used to treat urethral inflammation (Ihsan et al., 2018; Hosseini et al., 2019; Abd Elkodous et al., 2020). Zinc oxide nanoparticles have a good antioxidant function in combination with other components (García-López et al., 2018). In recent years, the development of enzymology has provided an effective tool for the removal of reactive oxygen species, and some inorganic nanoenzymes with an inherent antioxidant capacity have also been developed as neuroprotective therapeutic drugs, among which CeO_2_ is the most promising (Kwon et al., 2018; He et al., 2020). Hirst et al. documented the anti-inflammatory properties of CeO_2_ nanoparticles for the first time in 2009, as these nanoparticles inhibited the expression of iNOS in LPS-induced macrophages (Hirst et al., 2009). Soh et al. prepared a cerium oxide-zirconia compound nanoenzyme and found that it eliminated ROS production to inhibit sepsis (Soh et al., 2017). Therefore, inorganic nanomaterials with enzyme-like effects scavenge free radicals and exert anti-inflammatory effects by producing enzymatic reactions.

In addition, inorganic nanoparticles have good performance in fighting microbial infections. Gold nanoparticles improve the antibacterial activity of antibiotics through the targeted delivery of antibiotics (Patil and Kim, 2017). Meanwhile, the photothermal effect of gold nanoparticles irreversibly destroys the bacterial membrane structure and then kills bacteria (Hu et al., 2017). Silver, magnesium and iron particles, when reduced to nanosize, were suggested to exhibit antibacterial activity against *E. coli* and *S. aureus *(Yousefshahi et al., 2018; Videira-Quintela et al., 2020). Nanosilver is widely used in the medical field because of its strong antibacterial activity, lack of drug resistance and safety. Silver NPs target the respiratory system and cell division of microorganisms that eventually result in cell death (Panáček et al., 2018). Copper nanoparticles have a similar antibacterial mechanism in urinary tract infection (Al-Enizi et al., 2018). Titanium dioxide nanoparticles have also been used to destroy bacterial cells (Zheng et al., 2018). The antimicrobial activity is based on the photocatalytic property of TiO_2_ NPs (Guo et al., 2019). The production of reactive oxygen species (ROS) by TiO_2_ was also reported (Yousefshahi et al., 2018). In addition to their direct anti-infective effects, nanoparticles can also be loaded with antibiotics. Biocompatible Fe_3_O_4_ nanoparticles increase the efficacy of amoxicillin against gram-positive and gram-negative bacteria through magnetic targeting (Lu et al., 2017). Sulfur nanoparticles enhance the killing of urethral pathogens by delivering antibiotics (Paralikar et al., 2019). Notably, the metabolism of inorganic nanoparticles remains controversial, especially those containing heavy metals, which have the risk of metabolic toxicity (De Matteis, 2017). These nanomaterials have important application prospects in the treatment of urethral infectious inflammation.

#### Organic Nanomaterials

In the field of medicine, hydrogels have great potential for development. The structure determines the properties, and the biocompatibility, biodegradability and nanometer compound properties of hydrogels are commonly used in the medical field (Fuchs et al., 2020). Therefore, hydrogels are widely used in the medical field as drug release carriers and corneal contact lenses, in bone tissue and soft tissue regeneration, and in reconstruction and burn treatment (Chen et al., 2019). Sun Xiaoyong conducted clinical trials, and patients with prostatitis were divided into two groups, a group treated with terazosin hydrochloride combined with levofloxacin, another group treated with nanosilver hydrogels and silver nanoparticles through the anal route, and premature ejaculation grading and erectile function index were evaluated in the two groups of patients before and after treatment to assess sexual function and quality of life (Sun et al., 2019). He observed improvements in these indicators in patients treated with the nanosilver hydrogel (Sun et al., 2019). Although hydrogels have good sustained release and anti-inflammatory effects, chemical cross-linking reagents are often needed.

In addition to hydrogels, organic nanoemulsions are also commonly used as drug carriers. PLGA nanoparticles show excellent antibacterial properties against urethral pathogens by delivering trimethoprim (Brauner et al., 2020). Liposomes are widely used in biomedical research, especially in nucleic acid delivery research. Zhao et al. reported that the in vivo delivery and expression of hBD-2 via liposomes reduced mucosal damage, interstitial edema and inflammatory cell infiltration in animal models of UTI (Zhao et al., 2011). Active peptide nanoparticles have also been used in prostate therapy. According to Cao et al., nanoparticles coupled with the autoantigen peptide T2 display improved efficacy against CP/CPPS, which would improve the treatment approach (Cao et al., 2019).

In recent years, biologically derived nanomaterials, including extracellular vesicles, have been widely used in the field of biomedicine (Fan et al., 2019; Fan et al., 2019; Jiang et al., 2020; Wang et al., 2020). Extracellular vesicles are phospholipid bilayer membrane vesicles that are released by cells and transmit information between cells. They also play an important role in regulating inflammation in the body. Extracellular vesicles derived from neutrophils exert an anti-inflammatory effect because they express inflammatory cytokine receptors that bind to and clear inflammatory cytokines (Gao et al., 2017; Li et al., 2020). Researchers found that extracellular vesicles from other cellular origins also exert anti-inflammatory effects (Liu et al., 2020; Gao et al., 2021). Extracellular vesicles derived from mesenchymal stem cells inhibit inflammatory phenotypes by regulating immune cell signal transduction in individuals with chronic prostatitis (Peng et al., 2021). In addition, bionic extracellular vesicles are widely used in anti-inflammatory and anti-infection research. Jiang et al. achieved endotoxin and exotoxin cleanup and antimicrobial effects by constructing hybrid bionic extracellular vesicles targeting bacteria (Jiang et al., 2021). These studies provide new insights into the treatment of urethral inflammation.

### Nanomaterials for the Diagnosis of Prostatitis

Nanomaterials have been widely used in biosensors, molecular diagnosis, medical imaging and other research fields (Liu et al., 2014; Deng et al., 2017; Liao et al., 2018; Liu et al., 2019; Liu et al., 2020) and have wide application prospects in the diagnosis of prostatitis (Qindeel et al., 2021). In particular, in medical imaging, nanomaterials have been used as contrast agents to guide the treatment of prostatitis. Contrast agents enter the body through surface coupling or encapsulation in nanoparticles, which increase the acoustic reflectivity and form clearer images with increased brightness (Wu et al., 2019; Fan et al., 2021). Magnetic resonance (MR) produces an image of resonance signals caused by radioexcited external magnetic fields based on the spin of protons. MNPs have been used as contrast agents to modulate the undulation of T2 of water molecules to form the “target-MNP” polymer. At this point, MNPs and target molecules form a magnetic cluster through the specific binding of high-affinity ligands, resulting in faster attenuation of the NMR signal or a shorter transverse relaxation time (Polackwich and Shoskes, 2016). Compared with GMP, MTJ and μHall sensors effectively shorten the time required to complete immunoassays [58]. Computed tomography (CT) uses X-rays to create cross-sectional and three-dimensional images of different tissue decay states. The CT contrast medium plays a key role in distinguishing similar attenuation coefficients. Two types of CT contrast agents are composed of nanoparticles. One is an iodine-based nanosynthetic drug in which nanoparticles act as carriers of iodine (Xu et al., 2019), such as liposomal iodine [59]. The second category is metal-based contrast agents, which are composed of nanoparticles derived from various metals with high X-ray attenuation factors, including gold and zirconia. Nano-CT contrast agents are widely used in biomedical imaging. For example, gold nanoparticles are engulfed by red blood cells to form blood flow images (Han et al., 2019). CT pulmonary angiography is a minimally invasive angiography technique that rapidly infuses an iodine contrast agent into the pulmonary artery through the superior vena cava, the right atrium and the right ventricle through the superior vena cava and then to the pulmonary artery. Scanning using spiral CT or electron beam CT has been used as a first-line clinical screening method for acute pulmonary embolism.

With the rapid development of biomedical imaging technology in the 21st century, this technology has become an important method for the clinical diagnosis and detection of prostatitis. The field of biomedical imaging expanded from the initial X-ray imaging to magnetic resonance imaging (MRI), computed tomography (CT), and ultrasound used today after a long period of exploration and growth. Although these imaging techniques have different imaging principles, they all observe tiny lesions in a noninvasive manner, providing excellent images of humans due to their unique advantages. However, they have some inherent limitations. For example, magnetic resonance imaging has an insufficient spatial resolution, leading to low sensitivity [46]. Therefore, many contrast agents have been developed to improve the contrast between normal tissue and prostate lesions and thus improve the diagnostic accuracy.

The cause of prostatitis is multifactorial, and the disease course is long. The most commonly used mode in clinical practice, rectal administration, may cause damage to intestinal mucosa due to unstable drug absorption. Therefore, the treatment of prostatitis requires good imaging performance, strong compatibility, a high universality of imaging technology, and a high bioutilization of pharmaceutical preparations. Advances in nanotechnology have facilitated the development delivery systems to overcome prostate-related disorders. Advantages of nanocarrier preparations include the combination of a variety of drugs, including biomacromolecule drugs; reduced degradation of unstable drugs and slow and controlled release; and increased residence time of relevant drugs to avoid frequent injections to meet the needs of prostatitis treatment (Thakur and Agrawal, 2015). In addition, an increasing number of nanomaterials have attracted attention due to their excellent imaging performance. Currently, many nanomaterials have been successfully developed as contrast agents for clinical use (Lu et al., 2017; Hu et al., 2018; Song et al., 2018). For example, iron oxide nanoparticles and manganese oxide nanoparticles are used as MRI contrast agents because of their unique magnetic properties (Waddington et al., 2020). Gold nanorods have been used in photoacoustic imaging (PAI) due to their unique surface plasmon resonance properties (Huang et al., 2019). Surface engineering modification is often performed to maintain or improve their biocompatibility, colloidal stability and disease targeting and to achieve the more effective use of nanocontrast agents (Fan et al., 2021). Zhao Meng prepared a series of inorganic nanoparticles with a uniform morphology and imaging performance using polyglycol for ligand exchange to improve the colloidal stability and biocompatibility of the nanoparticles (Zhao et al., 2020). Then, the inappropriate groups were modified with spermidine, and finally, the targeted nanocontrast agent based on supramolecular chemical surface modification was obtained. Various methods were used to measure its properties, and the prepared contrast agents displayed good dispersiveness, colloid stability, and targeting, and the surface modification method was universal [51]. Surface engineering modifications based on supramolecular chemistry provide a new design idea and experimental basis for the future design and development of targeted prostatitis-related nanoagents. MR has the advantages of a high soft tissue resolution and no ionizing radiation, and thus it could be used in the diagnosis of prostatitis.

### Nanomaterials for the Prevention of Prostatitis

The key to preventing and controlling infectious diseases is to control the source of infection, cut off the transmission route and protect vulnerable groups (Nii-Trebi, 2017). Nanoantibodies can eliminate pathogenic microorganisms in animals, control the source of infection or cut off the transmission route to prevent diseases and protect people. *Campylobacter* infection is one of the most common foodborne infections in humans, and broilers are the main source of *Campylobacter* infection (Kelly et al., 2017; O'Brien, 2017). Nanoantibodies specifically target the outer membrane proteins of *Campylobacter jejuni* and *Campylobacter coli* in broilers, inhibit the fixed value of *Campylobacter jejuni* and block bacterial transmission (Vanmarsenille et al., 2017).

Passive immunity refers to the provision of pathogen-specific foreign antibodies to susceptible populations to achieve rapid protection in the short term. Traditional monoclonal antibodies are derived from the serum of humans or immunized animals, the manufacturing process is complicated, and the cost is high. Some animal-derived monoclonal antibodies easily cause adverse reactions. Nanoantibodies have become an alternative to existing passive immune antibodies. Many pathogens and external harmful substances enter the human body through the gastrointestinal mucosa. Vaccines targeting the mucosal surface can induce a mucosal immune response and prevent gastrointestinal infection. Oral vaccines are the most attractive route of treatment. However, vaccine antigens in the intestine often fail to reach potential immune-inducing sites, leading to a poor immune response. Aminopeptidase N (APN) is a receptor expressed on small intestinal cells and antigen-presenting cells (APCs). The combination of APN-specific targeting drugs with vaccine antigens significantly stimulates the immune response in the intestinal mucosa. Bakshi [53] constructed an anti-porcine APN nanoantibody with the Fc domain of conventional antibodies to form a bivalent fusion protein that triggered the intestinal IgA response after oral administration and confirmed the potential of vaccine antigen carriers (Wu et al., 2020). Modern bioengineering technology can help construct a variety of expression systems for nanoantibodies, improve the biosafety of nanoantibodies and promote their popularization and application. Rotavirus is the main cause of severe diarrhea in infants and young children, and specific therapeutic drugs are still lacking. Researchers have constructed expression systems in yeast, lactobacillus and transgenic rice to produce anti-rotavirus nanoantibodies that prevent rotavirus-induced diarrhea (Vandervaart et al., 2006; Martín et al., 2011). Transgenic rice were consumed by mice to absorb the nanoantibody expressed and stored in rice and to subsequently prevent diarrhea. These measures all suggest that nanoantibodies can be used as a complement to current vaccine-based infectious disease prevention measures (Tokuhara et al., 2013).

### Nanomaterials for the Treatment of Prostatitis

Anti-inflammatory and antimicrobial agents are the two main strategies for the treatment of urethral inflammation. We will summarize the application of nanomaterials in the treatment of prostatitis from antibacterial and anti-inflammatory aspects.

#### Application of Antimicrobial Nanomaterials in Prostatitis

Pathogenic microorganisms such as viruses, fungi and bacteria cause prostatitis, among which bacterial infection is the main pathogenic factor causing prostatitis. The number of antimicrobials used to eradicate type II chronic bacterial prostatitis is very limited. Treatment of CBP is hampered and challenging because most antimicrobial agents have a poor ability to penetrate infected prostate fluids and tissue (Charalabopoulos et al., 2003). Another reason is the lack of an active transport mechanism. Some drugs reach the prostate and achieve a minimum inhibitory concentration, but they also run the risk of bacterial resistance (Yu et al., 2018). Nanomaterials or nanoparticles may exhibit antimicrobial properties alone or enhance the efficiency of antibiotic administration. Antimicrobial NPs consist of metals and metal oxides, antimicrobial compounds, surfactant-based nanoemulsions and carbon-based nanomaterials. These nanoantibiotics may damage pathogens through several mechanisms: a) they may produce reactive oxygen species, damaging microbial cell components; b) they may degrade the cell walls of pathogens; c) they may interfere with energy transduction mechanisms; and d) they may slow or hinder DNA synthesis (Yoon et al., 2011; Kumar and Das, 2017; Fernando et al., 2018; Raza et al., 2019). Nanoantibiotics would be more useful in eradicating intracellular infections. While conventional antibiotics are effective at suppressing bacterial growth, they are least effective against bacteria that remain in quiescent cells. Urinary tract pathogens often take advantage of this limitation and cause urinary tract infections to recur after antibiotics have failed. Nanoantibiotics target residual bacteria in cells to avoid recurrence mechanisms.

Bacterial biofilms are an important barrier that promote bacterial self-protection and an important mechanism of therapeutic tolerance. Nanomaterials have shown unprecedented advantages in destroying bacterial biofilms. Li et al. realized the antimicrobial effect of the biofilm microenvironment response by designing antibiotic quantum dots (Li et al., 2020). In urethral infections, well-designed nanoparticles inhibited the production of biofilms, thereby inhibiting infection. For instance, Hosseini et al. reported that ZnO nanoparticles exert inhibitory effects on the biofilms of both isolates (Hosseini et al., 2018). These findings confirm the potential of zinc oxide as a treatment for catheter-associated urinary tract infections. In comparison, research into the antibiofilm effects of silver nanoparticles is more extensive and mature (Martinez-Gutierrez et al., 2013). Silver nanoparticles inhibit the formation of biofilms in organisms, including the natural marine environment (Fabrega et al., 2011), wastewater (Sheng and Liu, 2011) and mammals (Qin et al., 2014). The oxidation of silver ions is widely recognized as an antimicrobial mechanism. However, recent studies have shown that other mechanisms may exist. Saleh et al. found that Ag nanoparticles downregulated the expression of *Proteus novelis* and *Proteus vulgaris* fliL genes, which are clinically useful for urinary tract infections, thus exerting an anti-infection effect (Saleh et al., 2019). As in-depth research is conducted, the antibacterial mechanism of nanomaterials will be expanded, which will provide a more detailed basis for the antibacterial application of nanomaterials.

#### Induction of the Immune Response by Nanomaterials in Prostatitis

A large number of experiments have proven that the pathogenesis of prostatitis is closely related to the inflammatory microenvironment (Rees et al., 2015). However, the pathogenesis of prostatitis is complex, and the efficacy of monotherapy is limited. A treatment combining immunotherapy, antioxidant therapy and functional nanomaterials shows advantages. T2 is a specific peptide sequence isolated from the TRPM8 protein, which is encoded by prostate-specific genes (Miller et al., 2007), and has the ability to induce antigen-specific immune tolerance to antigenic peptides (Cheng et al., 2019). A prostatitis model was established in male C57 mice by intravenously injecting 0.2°mL of normal saline and 0.2°mL of a mixture of PLGA, PLGA-OVA and PLGA-T2. The PLGA-T2 group had a higher pain threshold, a lower frequency of urination and a significantly lower level of CPR than the other groups. Novel peptide T2-binding functional nanoparticles with autoantigens have been suggested to successfully alleviate or even cure prostatitis (Shandilya et al., 2020). Cao used antigen T2 combined with polyethylene-maleic anhydride-modified biodegradable PLGA nanotherapy, including the synthesis of biodegradable nanoparticles and conjugation to antigen T2 peptide, to induce immune tolerance in CP/CPPS mouse models (Cao et al., 2019). Mice treated with PLGA-PEMA-T2 showed increased pain thresholds, and reduced urination and prostate pathology. Compared with the other groups, serum levels of inflammatory mediators (TNF-α and CRP) were decreased and the level of the anti-inflammatory cytokine IL-10 was increased in the PLGA-PEMA-T2 group. PLGA-PEMA-T2 nanoparticles improved disease manifestations and upregulated IL-10 in mouse CP/CPPS models. The experiment confirmed the feasibility of using biodegradable nanoparticles combined with T2 antigen to treat prostatitis.

In addition, oxidative stress and inflammation are closely related to the immune responses that maintain homeostasis in the body. Oxidative stress is not only an important feature of inflammation but also a cause of inflammation (Czarny et al., 2018), (Liu et al., 2020). Selenium, a trace element in the human body, is a component of glutathione peroxidase and has the ability to inhibit the production of reactive oxygen species (Rao et al., 2019). In recent years, selenium nanoparticles bound to functional nanocomposites have developed rapidly. Yang, B-Y et al. eliminated oxidative stress after wound healing in the prostatic urethra following transurethral prostatectomy (TURP) using a multivoid Se@SiO2 nanosphere. A randomized beagle dog TUPR model was used to observe the level of oxidative stress during wound healing. Porous Se@SiO_2_ nanoballs promoted prostate urethral epithelial changes, enhanced the antioxidant capacity by inducing Ikk expression in macrophages, where I kappa B predominates, and p65 phosphorylation to inhibit oxidative stress and induce macrophages to differentiate into M2 phenotypes, reducing inflammatory reactions (Yang et al., 2019). Nanoselenium has been studied in combination with antibiotics for the treatment of urinary tract infections. El-Sayyad et al. synthesized gentamicin-assisted fungal-derived selenium nanoparticles under γ-ray irradiation to inhibit the resistance of urinary tract infection-causing pathogens (El-Sayyad et al., 2020).

## Summary and Outlook

The causes of prostatitis are complex and include pathogen infections, inflammation, free radicals, an abnormal immune response, sex hormone imbalance and so on. The treatment and diagnosis of prostatitis is facing great challenges. Nanomaterials with anti-inflammatory effects, such as CeO_2_, Fe_3_O_4_ and nano silver hydrosol, have been experimentally proven to be useful in the prevention, diagnosis and combined treatment of prostatitis. Although nanomaterials have achieved impressive results in experimental studies, their clinical conversion still faces significant obstacles. First of all, the metabolic pathway of some inorganic nanomaterials *in vivo* is not clear, and the cumulative toxicity is high. For example, silver nanoparticles, commonly used in urinary tract infections, accumulate in the body and cause liver and kidney toxicity. Secondly, traditional nanomaterials, as exogenous substances, are easy to trigger the immune response of the body, and are easily cleared by the immune system. In addition, nanomaterials as contrast agents also have the defects of low resolution and limited imaging depth. These problems greatly limit the application of nanomaterials in the clinical diagnosis and treatment of prostatitis. How to overcome the above obstacles has become the current research focus of nanomedicine.

The latest research progress summarized in this review, and it is not hard to find out the future research direction in this field. First, nanotechnology will promote the development of clinical diagnosis of prostatitis, especially molecular imaging research based on multimodal imaging technology will further improve the sensitivity and specificity of diagnosis. At the same time, nanotechnology will also facilitate the development of liquid biopsies, which are called upon to combine body fluid detection with medical imaging. Secondly, nanomedicine will break away from the traditional nanomaterials to the clinical application, which mainly depends on the development of new organic or biological sources of nanomedicines. The emergence of natural nanocarriers, such as exosomes, eliminates the immunogenicity and metabolic risks of traditional nanomaterials, making their clinical applications possible. Third, nanotechnology combined with machine learning can help achieve multifunctional integration and personalized diagnosis and treatment. Nanomedicine will undoubtedly revolutionize prostatitis diagnosis and treatment.
